# An Adaptive Data Gathering Scheme for Multi-Hop Wireless Sensor Networks Based on Compressed Sensing and Network Coding

**DOI:** 10.3390/s16040462

**Published:** 2016-04-01

**Authors:** Jun Yin, Yuwang Yang, Lei Wang

**Affiliations:** 1 School of Computer Science and Engineering, Nanjing University of Science and Technology, Nanjing 210094, China; junyin.nust@gmail.com; 2 School of Computer Science and Technology, School of Software, Nangjing University of Posts and Telecommunications, Nanjing 210023, China; leiwang@njupt.edu.cn

**Keywords:** data gathering, sensor networks, compressed sensing, network coding

## Abstract

Joint design of compressed sensing (CS) and network coding (NC) has been demonstrated to provide a new data gathering paradigm for multi-hop wireless sensor networks (WSNs). By exploiting the correlation of the network sensed data, a variety of data gathering schemes based on NC and CS (Compressed Data Gathering—CDG) have been proposed. However, these schemes assume that the sparsity of the network sensed data is constant and the value of the sparsity is known before starting each data gathering epoch, thus they ignore the variation of the data observed by the WSNs which are deployed in practical circumstances. In this paper, we present a complete design of the feedback CDG scheme where the sink node adaptively queries those interested nodes to acquire an appropriate number of measurements. The adaptive measurement-formation procedure and its termination rules are proposed and analyzed in detail. Moreover, in order to minimize the number of overall transmissions in the formation procedure of each measurement, we have developed a NP-complete model (Maximum Leaf Nodes Minimum Steiner Nodes—MLMS) and realized a scalable greedy algorithm to solve the problem. Experimental results show that the proposed measurement-formation method outperforms previous schemes, and experiments on both datasets from ocean temperature and practical network deployment also prove the effectiveness of our proposed feedback CDG scheme.

## 1. Introduction

Wireless sensor networks (WSNs) are currently deployed for many applications, such as environmental monitoring, civil structure maintenance, military surveillance, and so on. In most of these kinds of applications, sensor nodes in the network are set to periodically report their sensed data (*i.e.*, readings) to a sink node (or remote base station) through intermediate nodes’ relay. Under such circumstances, energy efficiency becomes one of the dominating issues of this data gathering process. Many solutions have been proposed based on various aspects, which include, among others, topology control (e.g., [1]), sleep scheduling (e.g., [2]), mobile data collectors (e.g., [3]), and data aggregation (e.g., [4,5,6,7]). The first three approaches focus on the energy efficiency of data gathering protocols or strategies, while the last one aims at reducing the required number of data packets to be sent to the sink node by eliminating data redundancy [8], hence it complements the others.

Generally speaking, depending on the information that is needed at the sink node, existing data aggregation research falls into two categories: “functional” and “recoverable” [9]. The first one corresponds to cases where only some values of statistical function of the network sensed data (e.g., AVG, MAX, SUM) are required by the sink node. Alternatively, the second one is for applications where the sink node needs the set of entire network sensed data. Compressed data gathering (CDG), based on the theory of compressed sensing (CS, [10]) and network coding (NC, [11]), has recently been proposed as a promising “recoverable” scheme, it enables the sink node acquire the complete network sensed data in an energy-efficient manner, as well as balance the energy consumption among sensor nodes.

First of all, we make some definitions on the problem to be discussed. Given a WSN of *N* sensor nodes, each having a piece of reading $x_{i}\left(i=1,\;2,\ldots ,\;N\right)$ at each data gathering epoch, we denote $X_{N}={\left[x_{1},\;\ldots ,\;x_{N}\right]}^{T}$ as the network sensed data. In a typical CDG scheme, the sink node will reconstruct $X_{N}$ by collecting only M (M≪N) weighted sums of $x_{i}$ from the network rather than all *N* original readings (in CS theory, these *M* weighted sums $Y_{M}=\;{\left[y_{1},\;y_{2},\cdots ,\;y_{M}\right]}^{T}$ are formed by $Y_{M}=\Phi X_{N}$, where the *M*-by-*N* matrix *Φ* is the measurement matrix (or called projection matrix), and each $y_{i}$ is the corresponding measurement (or projection) result of $X_{N}$; unless otherwise specified, “projection” and “measurement” are interchangeable hereunder). Such a reconstruction process is guaranteed and performed based on an observation fact that, in most network deployment cases, $X_{N}$ has a property called *K*-sparse (K≪N) under certain orthogonal transform domains (e.g., discrete wavelet transform, discrete cosine transform [10,12]).

**Figure 1:**
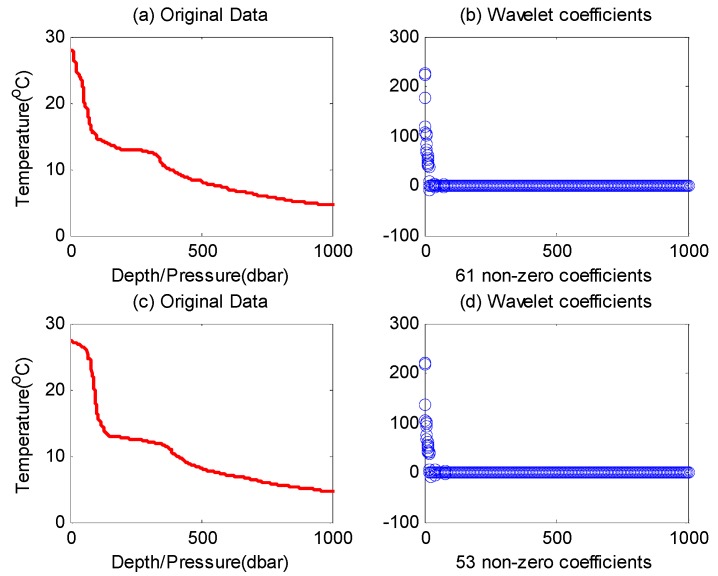
The sparsity varies in OTD datasets. (**a**) The Pacific Ocean temperature observed on 29 March 2014 (OTD A); (**b**) The corresponding coefficients of OTD A after six-layer Daubechies2 wavelet transformation; (**c**) The Pacific Ocean temperature observed on 2 April 2014 (OTD B); (**d**) The corresponding coefficients of OTD B after six-layer Daubechies2 wavelet transformation.

**Figure 2:**
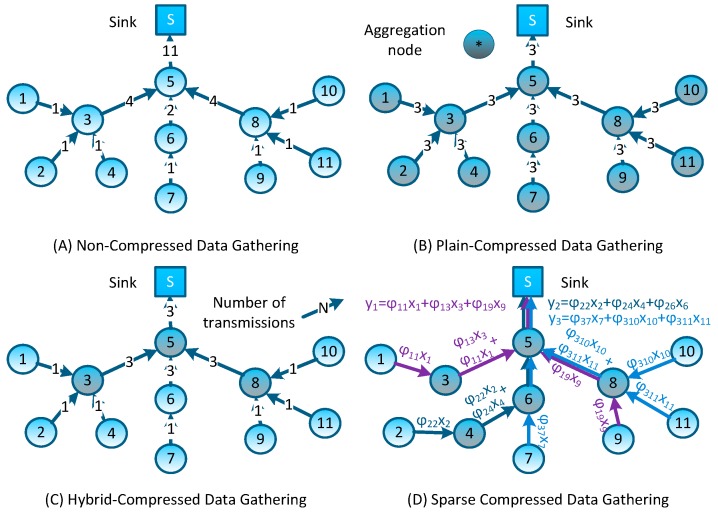
Non-CDG and typical CDG schemes.

In most previous CDG studies, classical CS theory is directly adopted without concern for the the WSN specialty, which inevitably incurs several shortcomings. For example, when processing a signal, CS theory conventionally supposes that the sparsity (*i.e.*, sparse degree) of this signal is fixed, while in a more general network deployment environment, the sparsity of the data observed by the network may change frequently. As shown in Figure 1, two 1000-reading datasets of ocean temperature data (OTD) are plotted with red lines. These datasets were collected from the Pacific Ocean on 29 March 2014 (Figure 1a) and 2 April 2014 (Figure 1c), respectively [13].

By comparing these two figures, we can see that the sparsity is changing; specifically, there are 61 larger coefficients after transformation of the first dataset (61-sparse signal), and only 53 in the second (53-sparse signal). There are also some other practical shortcomings, which will be elaborated in Section 3. To address these challenges, we propose an adaptive CDG scheme in this paper. During each data gathering epoch, we evaluate the current network sensed data at the sink node and adjust the measurement-formation process according to this evaluation. By doing so, it forms a kind of feedback-control process, and the required number of measurements is tuned adaptively according to the real-time variation of data to be gathered.

From another point of view, despite CDG’s ability to reduce the global communication cost, multiple studies show that the effectiveness of CDG is still affected by the strategy of the measurements’ formation process [9,12,14]. Several optimization schemes, such as Hybrid-CDG [9], are proposed to form all of the measurement results through a single routing tree in each data gathering epoch. To further reduce the energy consumption of such process, different from Hybrid-CDG, we supply a measurement-formation algorithm in this paper, where each measurement-formation path is treated individually in a data gathering epoch. Similar idea was also adopted by PB-CDG [14], yet the novelty of our approach lies in the path-generation procedure and the underlying method of measurement coefficients’ distribution, which omits the massive coordination among sensor nodes in the network.

The remainder of this paper is organized as follows: in Section 2 we first give a comprehensive overview on the typical CDG scheme mentioned above. Then, we propose our explicit motivation and main resolve method in Section 3. Next, we illustrate our data gathering approach in detail in Section 4 and Section 5. Simulation and practical experiment results are presented in Section 6. At last, we address the conclusions in Section 7.

## 2. Related Work of Compressed Data Gathering

In non-aggregation data gathering schemes, data packets generated by sensor nodes are directly forwarded to the sink node through a certain topology organization (e.g., the tree-type topology as shown in Figure 2A). These data gathering schemes do not exploit the correlation of network sensed data, resulting in the network forwarding a larger number of original packets; what’s more, in addition to sending their own detected data, nodes that are closer to the sink node tend to relay a number of packets from remote nodes (e.g., in Figure 2A, node #5 forwards 11 packets while each leaf node only forwards one packet). Such an imbalance of energy consumption will inevitably lead to the quick failure of the whole network. Thus, the lifetime of nodes which are closer to the sink node forms a bottleneck in these data-gathering schemes.

Plain-CDG, as shown in Figure 2B, is the earliest and the most rudimental data gathering scheme which exploits the CS and NC theory. During each data gathering epoch, every node needs to forward a fixed number of packets (denoted as *M*) to form *M* weighted sums $Y_{i}$ (we let *M* = 3 in Figure 2B, and $Y=\varphi_{i1}x_{1}+\varphi_{i2}x_{2}+\cdots +\varphi_{i11}x_{11},i=1,2,3$ , where $x_{j}$ is the reading of node #*j* and $\varphi_{ij}$ is its coefficient of this measurement). Note that each weighted sum corresponds to one measurement of network sensed data, and after receiving these measurement results, the sink node can reconstruct the network sensed data by adopting an appropriate CS reconstruction algorithm. Comparing to the Non-CDG schemes, Plain-CDG can reduce the number of transmissions, and balance the transmission load among sensor nodes.

The authors in [9] proposed a scheme based on Plain-CDG, called Hybrid-CDG (shown in Figure 2C), to further reduce the number of transmissions. In the Hybrid-CDG scheme, nodes whose degrees are equal to or less than *M* will employ the Non-CDG to forward their readings and the others will employ the Plain-CDG scheme to generate *M* measurement packets. By comparing Figure 2C to Figure 2B, it is easy to find that the Hybrid-CDG scheme indeed reduces the redundancy transmission of the Plain-CDG (e.g., the leaf node #1 forwards three packets in Plain-CDG while only one packet in Hybrid-CDG). More efficient data gathering schemes using random sparse measurements were first introduced in [15] and developed in [14] as follows: at the beginning of each epoch, *M* projection nodes are selected randomly to collect *M* measurements (*i.e.*, each projection node collects one measurement), meanwhile, each projection node is assigned or generates a sparse vector $\Phi_{i}$ by itself. Then, projection node #*i* is obliged to inform all nodes whose coefficient $\varphi_{ij}\neq 0$ to report their contributions ($\varphi_{ij}x_{j}$) back. After receiving all segments of a measurement ($y_{i}$), the projection node sends the result to the sink node through the shortest routing path. An example of such measurement-formation process is illustrated in Figure 2D. To form the measurement result $y_{1}$, as the projection node, node #5 has randomly generated a sparse coefficient vector $\Phi_{1}=\left[\varphi_{11}\;0\;\varphi_{13}\;0\;0\;0\;0\;0\;\varphi_{19}\;0\;0\right]$, then, for these non-zero coefficients ($\varphi_{11}$, $\varphi_{13}$ and $\varphi_{19}$), it sends transmission requests to nodes #1, #3 and #9; these nodes reply to the requests by sending their contributions back (e.g., node #3 will reply $\varphi_{13}x_{3}$). Note that, similar to other CDG schemes, those packets can be merged into one single packet on their measurement formation path (e.g., packets can be merged on nodes #3 and #5 for forming measurement $y_{1}$—this packet-merging process can be regarded as a network coding process [16]). Similarly, other measurements are formed in the network and forwarded to the sink node. It is easy to see that these sparse measurement-based CDG schemes would outperform prior dense schemes, because the formation of each measurement only involves several nodes, and the rest of the nodes can still stay in an idle/sleeping mode to reduce energy consumption.

## 3. Motivation and Proposed Method Design

### 3.1. Motivation of the Study

In most existing CDG studies, the network sensed data is considered to have a known and fixed sparsity. Therefore, according to the CS theory, once a recovery algorithm is picked, an upper bound of required measurements (*O*(*Klog*(*N*/*K*))) is determined. However, this assumption is not practical, and even problematic. Firstly, as depicted in Section 1, the sparsity of data signals observed in a real natural environment is changing, making it impractical to know the exact sparsity value before reconstruction. Secondly, to successfully reconstruct the network sensed data, the necessary number of measurements is continuously varying over a range, thus, taking a fixed upper bound as the number of measurements may inevitably cause excessive measurements. To show this point, we carried on an experiment to reconstruct an *N*-dimensional *K*-sparse signal from two typical measurement methods, namely a Gaussian measurement method and a Bernoulli measurement method (each entire measurement takes values of either ±1 each with probability *s*/2, or 0 with probability 1 − *s* (0 < *s* < 1)). We randomly generated an original signal *x* (*N* = 100, *K* = 10, *s* = 1/2), and to avoid the variations we repeated experiments of each measurement method for 600 times and employed the same algorithm (L1-formulation matching pursuit) on each reconstruction of *x*.

**Figure 3:**
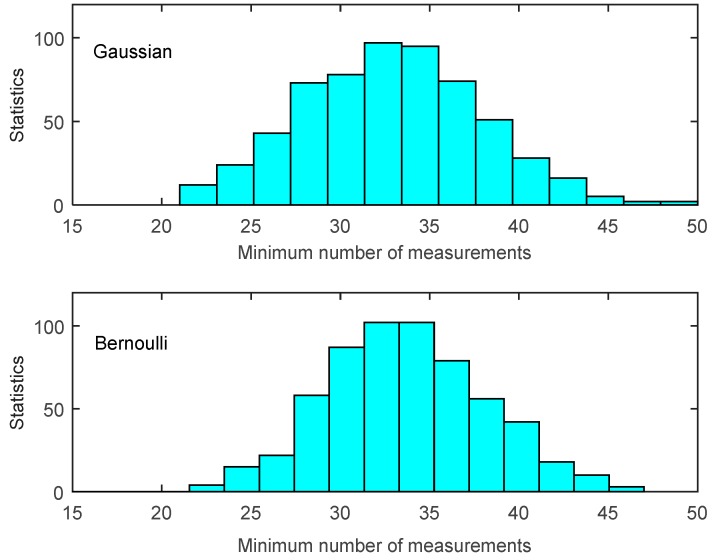
Histogram of the minimum times distribution of Gaussian (dense) (**top**) and Bernoulli (sparse) measurement for a 10-sparse signal (**bottom**).

The experimental results are depicted in Figure 3. We can conclude that both measurement ensembles exhibit high variance (mean values are 32.9450 and 33.8167, respectively; standard deviations are 4.8468 and 4.9414, respectively) and the most minimum number of required measurements are lower than the upper bound (50). This explains that schemes relying on an upper bound can guarantee successful data recovery, but it often means that many unnecessary measurements are taken (in our experiment 17.5 and 16.18 of 50 on average). For CDG applications, as shown in Figure 2, the formation of each measurement result requires processing and forwarding through several intermediate nodes; thus, reducing the unnecessary measurements will definitely decrease nodes' energy consumption.

Note that, comparing to the dense measurement method, we are particularly interested in the sparse measurement method. The reason is the sparse measurement method requires a very similar number of dense measurements (as shown in Figure 3), while for CDG applications, sparse measurement coefficient vector leads to a fewer interested nodes (interested nodes are the nodes whose corresponding coefficient of such measurement is non-zero) than that of dense method in the formation of each measurement, and thus other nodes can keep sleeping to save energy. For the sparse Bernoulli example in Figure 3, we experimented with *s* = 1/2, thus there were averagely 50% fewer nodes in the formation of each measurement than that of dense Gaussian method.

### 3.2. Main Program of the Proposed Method

**Figure 4:**
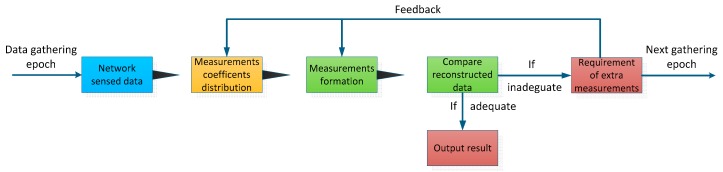
Main procedure of the proposed scheme.

In the CDG scheme, the key problem is how these measurements will be formed and sent to the sink node in an energy-efficiently manner. As we mentioned above, in actual network deployment, network sensed data is in fluctuation, and thus, the sparsity also changes with time. Given that the required number of measurements (*M*) cannot be decided at the beginning (it is up to the sparsity of the network sensed data *X*), it is inappropriate to use a specific upper bound in previous researches. In order to make up this defect, we propose a feedback control strategy without the requirement of having the specific sparsity knowledge of the network sensed data. The procedure is as follows (as shown in Figure 4 and Algorithm 1). During each epoch, the sink node evaluates the reconstruction quality of measurements which have been received; if the reconstruction quality indicators have not been obtained, a feedback packet will be sent to inform the nodes that additional Δ*M* measurements are required. This measurement-reconstruction process will repeat until the reconstruction quality is satisfied. Therefore, unlike existing schemes, a typical feature of our scheme is that the measurement procedure during each epoch is separated into several rounds. Since the network sensed data is adaptively and progressively reconstructed in our scheme, the sink node can also satisfy the varying levels of quality requirement of network sensed data by different users.
**Algorithm 1:** Main Procedure of the Proposed Scheme**Input:** Reconstruction quality requirement, time of each data gathering period *T***Output:** Network sensed data *X* in epoch *T_i_*1 Initiate the network settings, flag = 12 **If** clock interrupt of *T_i_* is received **do**3 generate feed-back packet and propagate it to the network4 Δ*M* measurements are propagated to the sink node through designed routing architecture5 the sink node receives and adds these projections into projection pool and gets reconstruction result $\hat{X}$6 evaluate $\hat{X}$7 **If** stopping rule is achieved then8 flag = 09 **Ifend**10 **While** flag = 011 **Ifend**12 **Return**
$\hat{X}$


## 4. Measurement Formation Process

According to the description in Section 2, CDG schemes require a certain number of measurements of network sensed data, and these measurement results are generated and aggregated on measurement-formation paths (*i.e.*, trees). As each measurement is a linear combination of multiple contributions, each of which is generated by multiplying the reading of an interested node and its corresponding measurement coefficient, an underlying question surfaces that how the measurement coefficients are generated and distributed. For the example proposed in Figure 2D, before responding to the requirement of the formation of the measurement $y_{1}$, interested nodes #1, #3 and #9 should receive their coefficients first. One may think that the sink node can pack and broadcast each measurement coefficient vector $\Phi_{i}$ to the network during each data gathering epoch, and every node in the network can directly acquire its measurement coefficient vector by monitoring these packets. However, for a WSN which has dozens of sensor nodes deployed in a large harsh field, such a process will incur a very heavy overhead of the time slice. What’s worst, such broadcasting requires the synchronization among nodes in the network, thus it is impractical, especially for those WSNs applying sleeping mechanisms.

**Figure 5:**
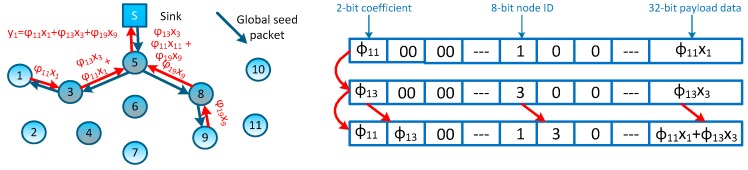
Global seed distribution and measurement result packet merge on node #3.

Considering the tree-type and sink-rooted measurement-formation path which covers all interested nodes, we propose to generate the measurement coefficients during constructing these paths. We achieve this object by associating a pseudo-random generator, which is an algorithm publicly known by both the sink node and sensor nodes in the network. In each measurement-coefficient-generating procedure, the sink node first produces a global seed corresponding to this measurement (in fact, the sink node’s running time would be a greater candidate for this global seed). Once the interested nodes in the network receive this global seed, it can generate its own measurement coefficient by adding the node ID. The sink node can generate a sparse coefficient vector $\Phi_{i}$, where each element $\Phi_{ij}$ is randomly generated by using the group seed: (global seed $S_{i}$, node ID *j*). Thus, coefficients in $\Phi_{i}$ obey an independent and identical distribution (i.i.d.) and the sink node can programme the measurement-formation path according to $\Phi_{i}$. For the measurement $y_{1}$ proposed in Figure 2D, such measurement-formation process is depicted in Figure 5, and two contribution packets can be merged on node #3. Now, there’s still an issue remaining, *i.e.*, how to energy efficiently build an optimal measurement-formation path which covers all interested nodes. In other words, how to ensure that all interested nodes in the network can receive the global seeds efficiently, and meanwhile how to minimize the total transmission cost. In the following sections, we would like to present our measurement-formation method.

### 4.1. Construction of the Measurement-Formation Path (Tree)

**Figure 6:**
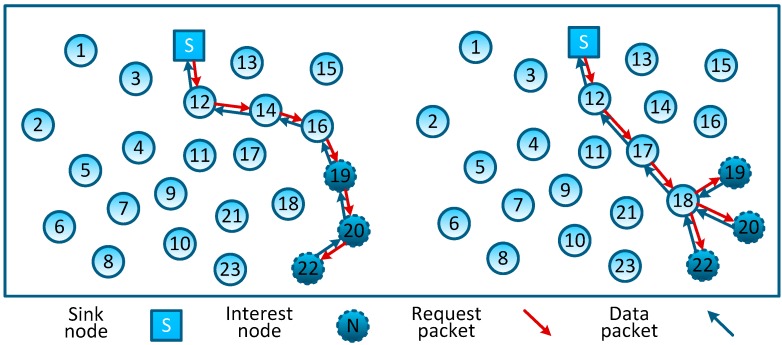
Measurement-formation path of PB-CDG and MLMS.

According to the perspective of graph theory, this measurement-formation process comes down to constructing a sink-rooted path which covers all interested nodes. To improve the efficiency of measurement formation, the total length of this tree should be minimized. Thus, the construction of such optimal tree is a typical Steiner minimum tree problem [17]. Unfortunately, it has been proved to be a kind of NP-complete problem [18]. To solve this problem, a heuristic method based on the All-Pair-Shortest-Paths (APSP) [14] algorithm has been proposed for CDG applications. However, APSP tries to form a line-shape measurement-formation path which does not consider the wireless transmission characteristics of WSNs. For example, the total lengths of both the left and right trees in Figure 6 are 6, but if we consider the eavesdropping characteristics of the wireless channels used in WSNs, the actual transmission overheads of the left and right trees are 6 and 4, respectively. From this example, we can find that the overhead of the construction of this tree depends on the number of non-leaf nodes, namely the number of relay nodes. Thus, this is a Maximum Leaf Nodes-Minimum Steiner Nodes (MLMS) problem, *i.e.*, we need to build a sink-rooted minimum Steiner tree (denoted by *T*) that covers all interested nodes (denoted by *I*). Note that not all interested nodes are located on the leaf nodes, some can also act as non-leaf nodes. MLMS tree has the requirement to minimize the number of introduced non-interested nodes (or called Steiner nodes), and maximum number of leaf nodes. In the next subsection, we will present the mathematical model of this problem, and after that, a global approximation algorithm is designed to solve this problem. Note that, classical converge-cast protocols can be a good candidate for data aggregation schemes as well as those CDG applications which do not consider the measurement coefficients generation/distribution (these CDG applications assume that nodes in network have already been assigned the measurement coefficients). Through this local gradient-routing mechanism, measurement results can be safely reported to the sink node. We proposed our MLMS tree-shape method based on an overall consideration of path generation, coefficients generation/distribution and the measurement results’ formation, and the measurement-formation path is created at the time of the global seed distribution.

### 4.2. Mathematical Model of MLMS Problem

We describe the WSN as a connected graph G=〈V,E,T〉, where *V* is the set of nodes; *E* is the set of undirected edges connecting any two nodes that can directly communicate with each other. $T=\left\{In_{i},S_{i},Y\right\},i=1,2,\ldots ,M$ is the set of *M* measurement-formation routing trees. $In_{i}$ is the set of interested nodes of *i*th measurement, and $S_{i}$ ($S_{i}\subset V-In_{i}$) is the set of Steiner nodes, $Y_{i}=\left\{y_{ij}^{t}\right\}$ is a binary variable indicating whether there is an edge between node *i* and node *j* for tree *t*, when (*i*, *j*) is the edge of the optimal Steiner tree *t*, $y_{ij}=1$, otherwise, $y_{ij}=0$. Then the objective and constraints of our MLMS construction problem can be formulated as below:

(1) $$\text{Min}\;z=\sum_{t=1}^{M}\sum_{(i,j)\in E}y_{ij}^{t},\;s.t.$$

(2) $$\sum_{i:(i,j)\in E}y_{ij}=1,\forall i\in I_{i},\forall j\in S_{i}$$

(3) $$\sum_{i,j\in I_{i}\cup S_{i}}y_{ij}=|In_{i}\cup S_{i}|-1$$

(4) $$\sum_{i,j\in I_{i}\cup S_{i}}y_{ij}=|In_{i}\cup S_{i}|-1$$

(5) $$\sum_{i\in I_{i},j\in I_{i}\cup S_{i}}y_{ij}^{t}=\left\lceil \frac{N}{M}\right\rceil ,\forall t\in T$$

(6) $$y_{ij}^{t}\in \{0,1\},\forall (i,j)\in E,\forall t\in T$$

Constraint (2) limits each interested node that can only connect with one Steiner node. By using Constraint (3), the degree of each Steiner node is limited to no less than 2. Constraint (4) ensures that the result we obtained is a spanning tree. Constraint (5) is a restriction on the number of interested nodes for the spanning tree.

### 4.3. Scalable Algorithm for MLMS Construction

Notice that all interested nodes *Iv* within the communication range of node *v* can be considered to come from the same group, and based on this circumstance, we can perform a greedy iterative algorithm to reduce the complexity of MLMS construction problem. Without loss of generality, node *v* can perform the iteration operation on behalf of other nodes in such a group. During each iteration, we first select the node that connects the most interested nodes or groups; combine this node and its associated nodes or groups to form a new group; and we repeat this process until find out all groups. Next, in order to make group leader nodes and the remaining interested nodes be connected with the sink node, an optimal Steiner tree approximation algorithm is required. Since this problem is NP-complete, we proposed an alternative algorithm which is built upon the minimum spanning tree (MST) algorithm. The scope to run the MST is the closure that takes the sink node as a fulcrum and contains all the remaining interested nodes. Then, we perform the Graham scan algorithm to obtain a convex hull of all remaining groups. At last, we perform the MST algorithm to connect these nodes in convex hull. Detailed description of this algorithm is shown in Algorithm 2. It is easy to see that for each measurement formation tree, maximum iterations times for group formation is *ρ*/2; and for the node-sparse graph, the proposed algorithm has a complexity of $O\left(\rho^{2}d^{2}\right)$, and for the edge-sparse graph is $O\left(\rho^{2}d^{2}+nlogn+n^{2}\right)$, where *d* is the average degree of the nodes in network, *n* is the number of edges and *ρ* is the number of interested nodes.
**Algorithm 2:** Construction of the Measurement-Formation Path**Input:** Sparse measurement coefficients vectors $\Phi_{1},\Phi_{2}\ldots ,\Phi_{M}$ each with *n* i.i.d. elements; nodes’ maximum communication range *R_comm_*;**Output:** Set of measurement formation a trees $T=\left\{T_{1},T_{2},\ldots ,T_{M}\right\}$, $T_{i}=\left\{In_{i},S_{i},Y^{i}\right\}$1 **For** each measurement i=1, 2,…, M2 Calculate the set of interested nodes $In_{i}=\left\{all\;nodes\;s.t.\;\Phi_{ij}\neq 0,\;j=1,\;2,\ldots ,n\right\}$.3 Calculate the set of neighbor nodes $Ne=\left\{each\;node\;v\;s.t.\;dist\left(v,t\right)\leq R_{comm},\;v\in V,\;t\in In_{i}\right\}$.4 **Do**5 Calculate the number of edges incident from Ne to $In_{i}$ (denoted as De{|Ne|}).6 **If**
Max(De{|Ne|})≥27 Remove all adjacent nodes of node *v* from $In_{i}$ where $v\in In_{i}$ and De{v}=Max(De{|Ne|})8 Add node *v* into *In_i_*9 Renew *Ne* and *In_i_*10 **Ifend**11 **While** ($\left|In_{i}^{new}\right|<\left|In_{i}^{old}\right|$).12 Calculate the convex hull (denoted as Cl) of set Ne∪{sink} by using Graham scan method13 Construct the MST which takes the sink as a root and connects all *Cl* nodes14 $T_{i}=\left\{In_{i},Cl,Y^{i}\right\}$ where $Y^{i}$ recorded the edge information of such MST15 **Forend**16 **Return**
$T=\left\{T_{1},\ldots ,T_{M}\right\}$


### 4.4. MLMS Tree Construction and Maintenance

**Figure 7:**
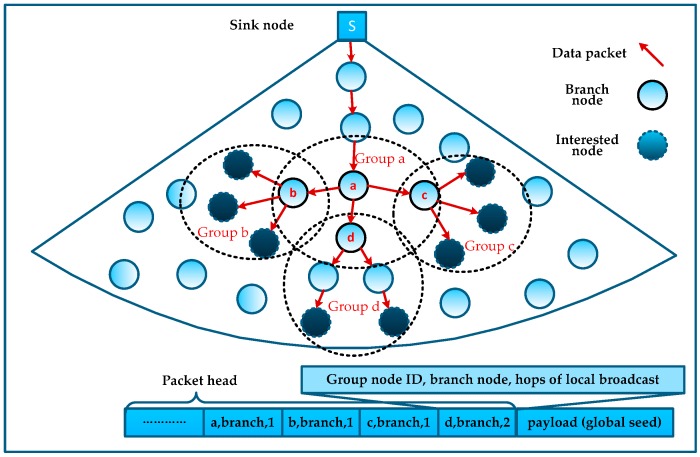
MLMS construction and the compressed packet format.

In order to avoid a large number of control packets caused by constructing MLMS trees, after calculating the approximate minimum cost MLMS path *T*, we let the sink node pack the information of *T* with the global seed into several packets. Through the nodes’ relay of these packets the MLMS tree is constructed orderly (as shown in Figure 7), but for a large-scale network (meaning there are many interested nodes in each measurement and the path from the sink node to the interested nodes may be extremely deep), the complete path information will take up a lot of space in the packet head. Due to the length limitation of packets transmitted in the WSN, the sink node needs to compress and encode the information of *T*.

Noticing that Algorithm 2 divides the neighboring interested nodes into a group, we can use the effect of wireless communication to locally broadcast such packets at each branch node and thus all its neighbors can overhear them. Thus, in the branches of the MLMS path, we only need to encode the branch node information and add the local broadcast hops to the packet header, rather than the information of all interested nodes. In this way, we can compress the path information to reduce the number of control packets.

As shown in Figure 7, all interested nodes and branch nodes are in four groups a, b, c and d, and we can guarantee that all interested nodes can receive the corresponding global seed simply by coding the information of branch nodes into the packet header, and the MLMS path can be constructed in disseminating such packets. Obviously, this mechanism is affected by the density of the interested nodes (*i.e.*, the proportion of the number of interested nodes to the total number of network nodes), and we will evaluate it in Section 6.

### 4.5. Maintenance of MLMS Measurement-Formation Path

WSNs are always deployed in a harsh environment, where unreliable wireless links and failure of certain nodes are prevalent, which potentially leads to the failure of creating and maintaining the MLMS path. In fact, the failure of any intermediate node compromises the delivery of all data aggregated and sent by the previous nodes in the path. Hence, some improvements to the scheme should be implemented. We will discuss this problem from two aspects: the nodes failure and the packets loss.

**Figure 8:**
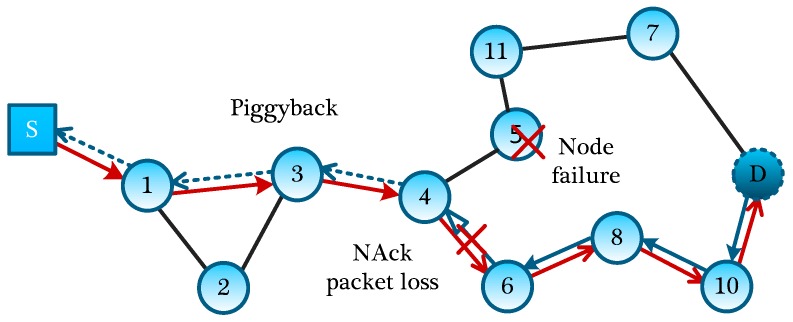
Piggyback nodes recovery and NAck packets loss.

Because the failures of other nodes will not affect the current MLMS measurement formation process, we mainly deal with the node failure on the MLMS path. If a node has failed, a new routing path should be established in a timely fashion, and the information of this failed node should be reported to the sink node to guide the building of another MLMS path. Then, two kinds of node failures on a MLMS path will be discussed below, including the group head node failure and the ordinary node failure. If a group head node at the end of MLMS path has failed, a new head node needs to be selected to continue to transmit packets of nodes in the group. Through dividing the remaining nodes into several small groups, we can choose the node which has the second-highest number of neighbor nodes to act as a new group head. If a node on the path fails to operate, its upstream node will choose the next neighbor nodes, which is similar to the GPSR protocol [19], and continue to transmit the packet, since all routing information (heading to interested nodes) is encoded into the packet head. As shown in Figure 8, when node #5 has failed, and its upstream node #4 encounters this situation, according to the working nodes in the neighbors, it will automatically select a next-hop node (e.g., node #6). But at this time, the routing path is probably not the optimal path, and if necessary, the optimal MLMS path should be recalculated.

For the node-failure message return, we need to respectively deal with node failures in different measurement-formation periods. In the distribution of the global seed, we can use the Piggyback method, which allowing the sender to add the ID of the failure node to the fixed position of seed-distribution packet. As node #4 sends packets to node #6, because node #3 can eavesdrop on the packet, it writes the ID of failure node #8 into a corresponding fixed position of its next packet to be sent, and in turn, until the failure information of node #5 reversely backtracking to the Sink through the normal data channel.

Therefore, without increasing any control packet, by adopting the eavesdropping property, information about failed nodes is quickly reported to the Sink, while for the information return of the failed nodes in the process of measurement-formation, we can directly write the ID of the failed node to the fixed position of the measurement-formation packet. With the return of measurement-formation packets, the sink node can directly obtain the information of failing nodes.

For each measurement formation process, both in the global seed distribution period and the establishment of measurement result, it will inevitably experience packet losses, and if the packet losses occur without a recovery mechanism, failure of this measurement formation will be induced, definitely. If a node in MLMS path discoveries that several packets in a sequence are not received within an acceptable TTL time, it will ask the upstream node for these packets along the reverse path. If the upstream node does not keep these packets, this process will continue until reaching the interested nodes. As shown in Figure 8, if node #4 fails to receive the packet from #6, node #4 will request this packet from its upstream node #6; if node #6 does not keep it, node #6 will request its upstream node #8 for this packet, and such process will be repeated until the packet is found. Of course, in order to further improve the reliability and recover the lost packets timely, we can set nodes around the MLMS paths to buffer the packets they have eavesdropped on; when certain packets are lost, they can be recovered quickly and accurately through multicast inquiry messages to the neighbor nodes. What’s more, a consultation method can also be used to further improve the reliability of packet transmission (for example, three-handshake negotiation). The disadvantages of these two methods are the large overhead of transmitting control messages, and we will evaluate it in Section 6.

## 5. Adaptive Termination Rule of the Measurement-Formation

As we mentioned before, after choosing a reconstruction algorithm (such as BP, or MP), the sink node can reconstruct the *K*-sparse original signal with the probability close to 1 as long as it receives a certain number of measurement results (theoretically, *O*(*KlogN*)). However, as shown in Section 3, this bound cannot be applied to our method directly, because it requires prior knowledge of the sparsity information of the network sensed data. By using the conclusion from “sequential compress sensing [20]”, an adaptive termination condition of measurement procedure was proposed in literature [21]. Specifically, assume that we can obtain measurement result of a *K*-sparse signal *x* (*x* ∈ ℝ^*N*^, *K* is unknown) step by step (each step gets one measurement result and we denote the measurement on step *i* as $y_{i}=\varphi_{i}x$); if the reconstruction result on step *M* is ${\hat{x}}^{M}$ and ${\hat{x}}^{M}={\hat{x}}^{M-1}$, then ${\hat{x}}^{M}$ is the result which we need (*i.e.*, ${\hat{x}}^{M}=x$) with probability 1.

Although this termination rule is very concise, it still has some limitations when applied it to CDG for WSNs. Because this rule is based on the dense measurement method (each measurement coefficients vector $\varphi_{i}$ obey Gaussian distribution), as shown in Section 2, each measurement formation will concern every node in the network, which will lead to more energy consumption than the sparse measurement method. On the other hand, coefficients in the measurement vector of this termination rule are continuous; thus, it is very difficult to generate on cheap WSN sensor nodes. Therefore, we need to find a termination rule for the sparse measurement method in this study. The difference emerging from the dense Gaussian measurement method is that the probability of being nonsingular for each submatrix of the measurement coefficient matrix is no longer 0. In order to meet our specific demands, we modified the termination rule in [20] to obtain agreement for a certain number of consecutive measurements (denoted as Δ*M*) (as shown in Proposition 1). Note that, we assume that the sink node can obtain and reconstruct the sparse Bernoulli measurement results step by step, and *x* represents the *N*-dimensional data vector which is desired by the sink node in each data-gathering epoch.

**Lemma 1.**  *Let v be an n-dimensional sparse Bernoulli vector (*$v\in {\left\{-1,0,1\right\}}^{n}$*) and* $\|v_{0}\|=\lambda$ (0<λ≤n)*. Let W be a deterministic w-dimensional subspace of* ${\mathbb{R}}^{n}(0<\lambda <w\leq n)$*, then* $Prob(v\in W)\leq 3^{w-n}$.

**Proof.**  Given *W* being a *w-dimensional* subspace, there exist *w* coordinates $c_{i1},\ldots ,c_{iw}$ that determine all the other *n-w* coordinates of an arbitrary vector $c=(c_{1},\ldots ,c_{n})\in W$. Thus, if we condition on the coordinates $c_{i1},\ldots ,c_{iw}$ of *v*, there is at most one case to make v∈W in the other *n-w* coordinates. Hence the probability of v∈W is at most $3^{-(n-w)}$.

**Proposition 1.**  *Suppose that the sink node has got* M−1 *measurement results in previous* M−1 *steps, and at step M, if the sink node have got the measurement result* $y^{M}$ *from the network, it can get a reconstruction result of network sensed data (denote as* ${\hat{x}}^{M}$*) from these received measurement results; if* ${\hat{x}}^{M}={\hat{x}}^{M-1}=\ldots ={\hat{x}}^{M-\Delta M}$*,* ${\hat{x}}^{M}$ *is the reconstruction result which we need with probability no less than* $1-3^{-\Delta M}$.

**Proof.**  We prove this proposition by using *argumentum ad absurdum*. We denote the measurement coefficient vector at Step *i* as $\varphi^{i}$, and the measurement result of Step *i* as $y^{i}$. Assume that, ${\hat{x}}^{M}={\hat{x}}^{M-1}=\ldots ={\hat{x}}^{M-\Delta M}$ and ${\hat{x}}^{M}$ is not the correct reconstruction result of *x*, then $x\neq {\hat{x}}^{M-\Delta M}=\ldots ={\hat{x}}^{M}$ is valid. Because we have obtained the solution result ${\hat{x}}^{M-\Delta M}$ at the Step M−ΔM, equation ${\left(\varphi^{M-\Delta M}\right)}^{T}{\hat{x}}^{M-\Delta M}=y^{M-\Delta M}={\left(\varphi^{M-\Delta M}\right)}^{T}x$ comes into existence. Thus, according to the assumption that ${\hat{x}}^{M-\Delta M}={\hat{x}}^{M}$, it is easy to obtain ${\left(\varphi^{M-\Delta M}\right)}^{T}(x-{\hat{x}}^{M})=0$. By following this idea, for ΔM measurement coefficient vectors generated from Step M−ΔM+1 to Step *M*, we can also get equations${\left(\varphi^{i}\right)}^{T}(x-{\hat{x}}^{M})=0,i=M-\Delta M+1,\ldots ,M$. Let *V* be a vector space which is spanned from the non-zero vector $\hat{x}-{\hat{x}}^{M}$, and if this equation set is correct, entire ΔM measurement coefficient vectors $\varphi^{i}$ must exactly belong to the orthogonal complement of *V* (denoted as $W^{⊥}$ where $W^{⊥}=\{x|x\in {\mathbb{R}}^{N}s.t.\;(x,y)=0\;for\;all\;y\in V\}$). For a 1-dimensional nonzero linear transformation $(x-{\hat{x}}^{M})’$, its orthogonal complement is an (n−1)-dimensional subspace of ${\mathbb{R}}^{N}$. Hence, from Lemma 1, it can be seen that the probability of an imprecise solution ${\hat{x}}^{M}$ being calculated and kept is greater than $3^{-\Delta M}$, as desired.

Proposition 1 provides us a termination rule of sparse Bernoulli measurement formation by comparing several construction results. In our experiments, signals are generated with the same signal length and sparse degree as shown in Figure 3. We test the signal reconstruction under the termination rule proposed in Proposition 1 with different Δ*M* values, and the experiment results are shown in Table 1. We can see that Proposition 1 presents a very efficient terminating condition in the reconstruction of sparse Bernoulli measurement method. The larger the quantity (Δ*M*) is adopted, the lower error reconstruction generates (*i.e.*, 128 errors happen under Δ*M* = 1, while only 2 under Δ*M* = 2). Thus, our data gathering process can adapt to the various requirements of network applications by adjusting the value of Δ*M*.

## 6. Numerical Results

We evaluate the performance of the proposed and existing schemes through both simulations and practical experiments. We separate our experiments into two main parts: (1) the performance simulation of the cost of MLMS measurements formation and (2) the reconstruction of sensed data in the network by using our proposed termination rule. We employ OMNeT++ to establish our sensor network with different randomly distributed wireless nodes. Due to the data reconstruction performance of the sink node involved in the sparsity of data itself and the chosen of reconstruction algorithm, it belongs to the category of the application layer; as a matter of convenience, we adopt MATLAB to reconstruct the data received by the sink node.

### 6.1. Energy Consumption of Measurements Formation in MLMS Construction and Maintainance

We randomly disperse *N* nodes in an 800 × 800 two-dimensional plane. Each node has the same transmission distance of *r*. Only the two nodes, whose Euclidean distance (*d*) between each other is less than *r*, can communicate with each other. In order to guarantee the network connectivity, the communication distance of nodes is fixed at $800\sqrt{5}/N$ [22], and the collection node is located at (400,800). The detailed parameters are shown in Table 2. We compare our MLMS method with the schemes mentioned in the Section 2, including Plain-CS, Hybrid-CDG, and the PB-CDG [14]. Measurement coefficients for all of these methods obey Bernoulli distribution (entries take value of either 1 or −1, each with probability of 1/4, and value 0, with probability of 1/2).

**Figure 9:**
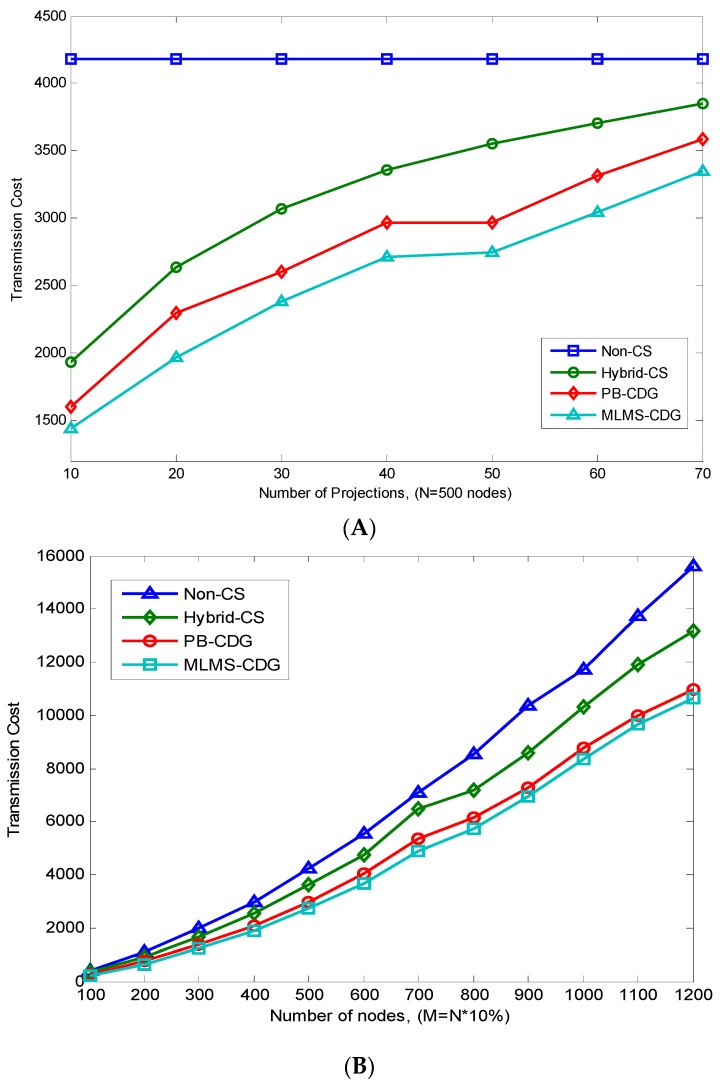
Comparison of MLMS measurement formation and other methods. (**A**) Experiment of the influence of the number of measurements; (**B**) Experiment of the influence of the density of the network.

We first test the energy consumption of various measurement formation methods with the increasing number of measurements and the nodes in the network (density). Note that, similar to reference [14], we measure the node’s energy consumption by the number of packets it transmitted. The experiment results are shown in Figure 9. We can see that Non-CS and Hybrid-CS incur higher energy cost than that of PB-CDG and the proposed MLMS method, because all measurements in these two methods are collected through a single fixed forwarding tree, which does not consider the sparse structure and derivative routing path of each measurement. Despite the fact that both MLMS-CDG and PB-CDG are derived from the idea of a Steiner tree, MLMS-CDG consumes less energy than PB-CDG (up to 14.52% and 17.76% in Figure 9A,B, respectively). This is largely because MLMS incurs lower overhead of measurement coefficient distribution than PB-CDG (with an average of 11.4% in different network densities).

**Figure 10:**
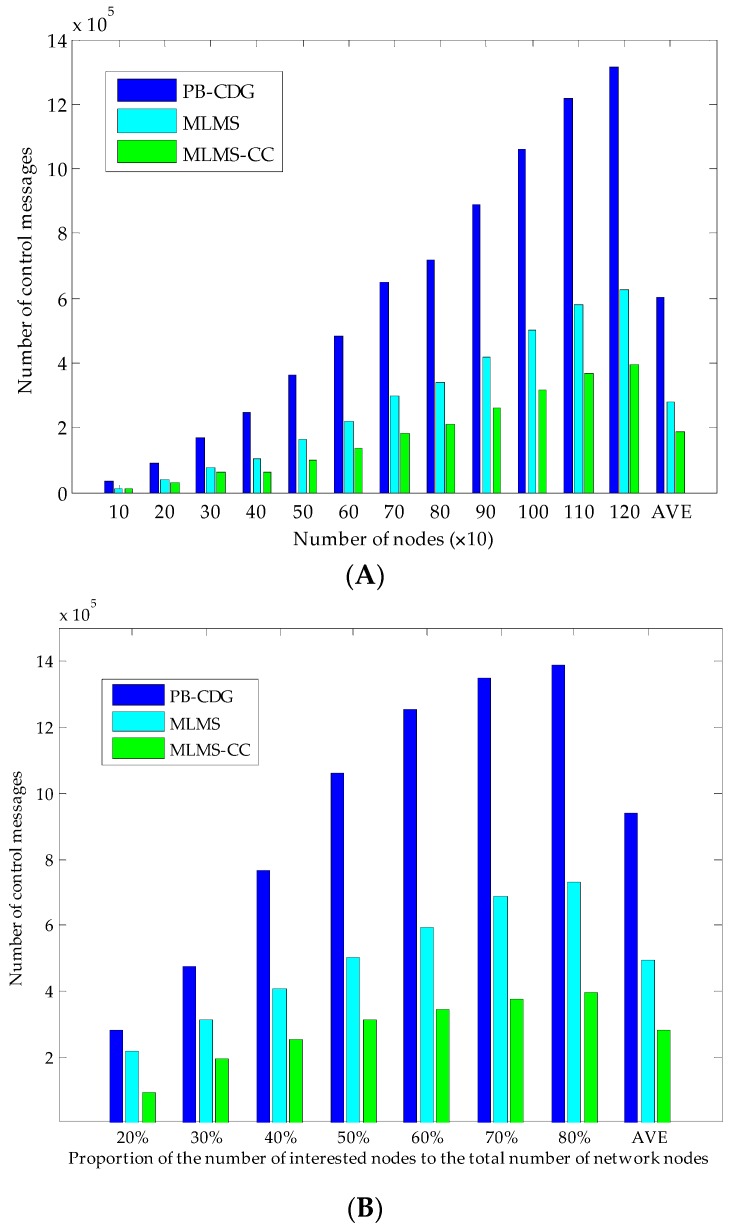
Comparison of the number of control messages of MLMS measurement formation and other methods. (**A**) Experiment of the overhead of the construction of measurement-formation paths (interested nodes of each measurement account for 50% of nodes in network); (**B**) Experiment of the influence of the number of interested nodes.

We then experiment on the number of control messages of PB-CDG and MLMS measurement-formation methods with the increasing value of the node density in the network. Because the PB-CDG method did not specify the generation method of measurement coefficients as well as the processing strategy of the node failures and the packet losses, we treat the PB-CDG scheme in the same manner of measurement generation as MLMS. Figure 10A shows the overhead of constructing the measurement-formation paths by using original methods and compressed construction method (MLMS-CC). With the growth of the number of sensor nodes, we observe that PB-CDG increases more sharply than both MLMS and MLMS-CC methods do. This is mainly because PB-CDG builds several line-shape paths for each measurement formation which will take a greater number of control messages in distributing global seeds (overheads of MLMS and MLMS-CC are averagely 53.33% and 68.70% less than that of PB-CDG). Then, we experiment on the influence of the intensity of interested nodes. As shown in Figure 10B, at the beginning of increasing the density of interested nodes, control messages of all schemes increase sharply, but the difference is that the MLMS-CC tends to increase slowly after intensity ratio equal to 50% which is less than MLMS (60%) and PB-CDG (70%). This is because if the interested nodes have accounted for a certain part of nodes in the network, continuing to increase the number of interested nodes will lead to a high efficiency of constructing groups in MLMS method, therefore the MLMS-CC and MLMS methods are not severely affected.

**Figure 11:**
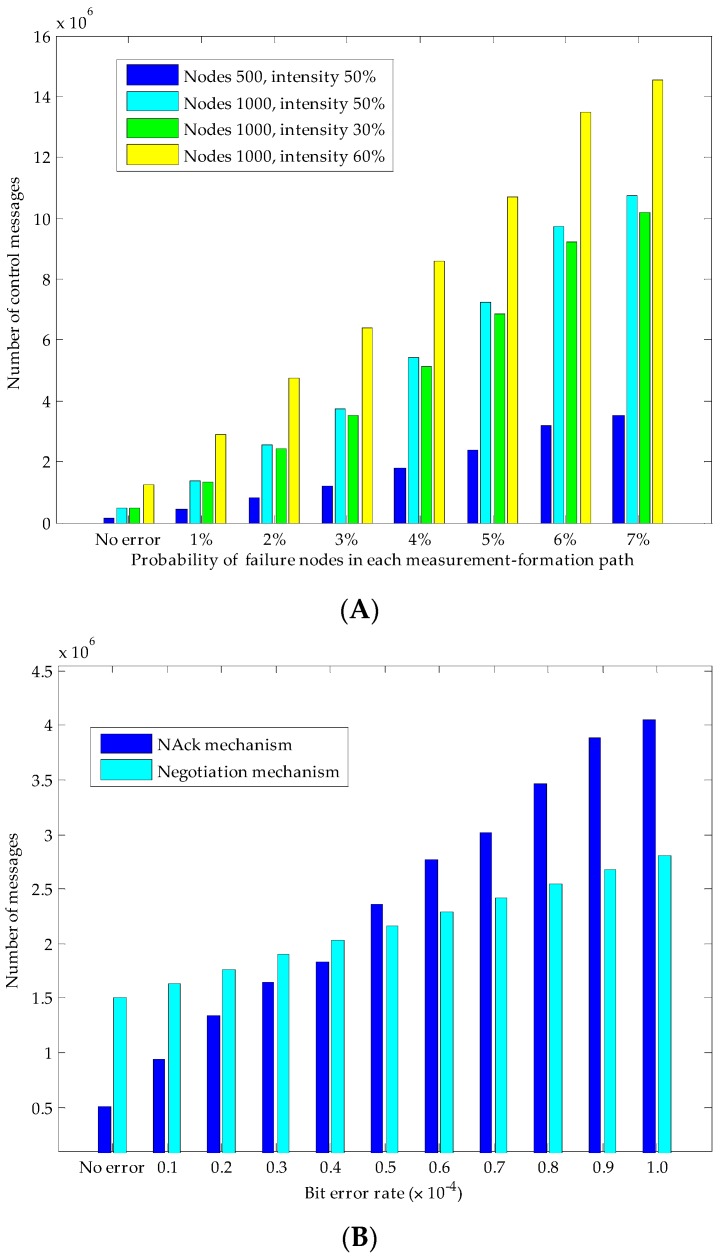
Overhead of maintaining measurement-formation paths. (**A**) Influence of nodes failure; (**B**) Influence of packets loss.

In the performance evaluation of using the Piggyback mechanism to process node failures, we respectively simulated the influence of network size (number of nodes 500 and 1000) and intensity of interested nodes (30% and 60% of nodes in the network). Each node on a MLMS path may fail with a probability of 1%~7% during the measurement-formation periods. The experiment results are shown in Figure 11A, we can see that both the network size and density of the interested nodes will have a greater impact on the Piggyback mechanism, but the impact of network size is significantly larger than that of the intensity of interested nodes (for example, under the condition of node failure rate = 7%, if the number of network size doubles (from 500 to 1000), the number of packets increases 205.46%, while if node density doubles (from 30% to 60%), the number of packets only increases 42.17%). This is because when the network size increases, it will inevitably lengthen the MLMS paths, which will result in a remarkable overhead increase in returning failure-node information in the Piggyback mechanism; however, increasing the density of interested nodes does not have a significant influence.

Because nodes in the network communicate over an erasure channel, by setting the bit error rate (BER) of this channel, we are able to generate a random packet losses context. We simulated the proposed NAck mechanism and the hand-shaking negotiation mechanism on a 1000-node network (the intensity of interested nodes is 50%). The experimental results are shown in Figure 11B. We can easily find the differences between these two mechanisms. With the increasing of the BER, packets transmission in the NAck mechanism increase more rapidly than in the negotiation mechanism. Specifically, when the BER is relatively small, using NAck mechanism will incur less number of control packets; when BER is around 0.4 × 10^−4^~0.5 × 10^−4^ (in this case, the packets successful delivery rate is about 95%), both mechanisms have a similar performance, and if we still increase the error rate, the negotiation mechanism will outperform the NAck. This means that, when the network conditions are poor, the negotiation mechanism will be a better choice.

### 6.2. Measurement-Formation and Reconstruction of OTD Datasets with the Proposed Termination Rule

In order to verify the effectiveness of the proposed adaptive termination rule, we select the two OTD datasets (depicted in Figure 1) as the original experimental signal. Due to the influence of Δ*M* on the performance of this algorithm, we carry out the experiment on the number of measurements and the quality of signal reconstruction under different Δ*M* conditions. With the purpose of ensuring the accuracy of the experimental results, we repeat the experiment of each set of parameters 500 times, and the detailed experimental parameters are listed in Table 3.

**Figure 12:**
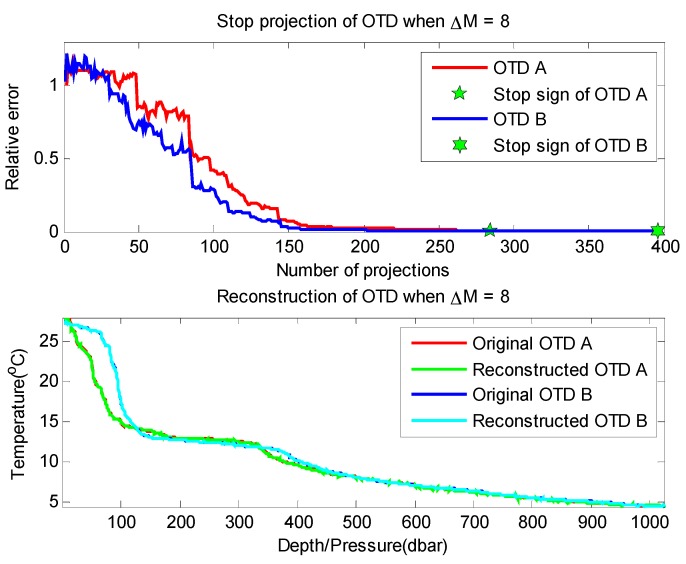
Experiment process (Δ*M* = 8) of our proposed method on OTD datasets. (**top**) Changing process of the relative error; (**bottom**) Reconstruction results.

**Figure 13:**
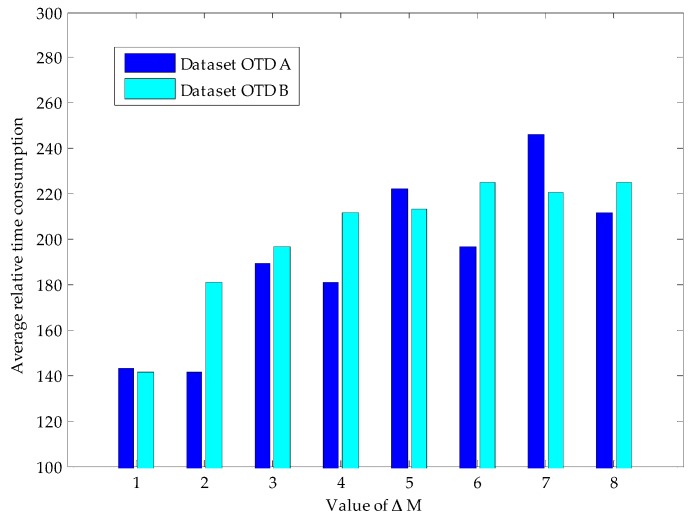
Experiment process (Δ*M* = 8) of our proposed method on OTD datasets.

Figure 12 shows an execution process (Δ*M* = 8) of our proposed method in the recovery of two datasets. We can find that in the reconstruction process of two datasets, the relative error between the reconstructed signal and the original signal tends to be 0 as the number of measurements increases, and this decline continues until reaching the termination condition of Proposition 1. It can be seen that when the algorithm terminates (dataset OTD A stops at Step 276, and OTD B stops at Step 380), we can acquire high quality reconstruction results (relative error values are 0.0068 and 0.0036, respectively). Detailed experimental results are shown in Table 4, and Figure 13 shows the average relative time consumption of the experiment on these two datasets.

By comparing the experimental results of these two time-continuous datasets, we find that the number of required measurements is different (*i.e.*, the number for OTD-B is less than that of OTD-A), which is due to their different sparsity degrees (as shown in Figure 1, the sparsity degree of OTD-B is higher than that of OTD-A, *i.e.*, 53-sparse and 61-sparse, respectively). With the increase of Δ*M*, the average number of measurements required by the two datasets will increase rapidly (*i.e.*, OTD-A increases by 106.5% and OTD-B increases by 59%). The minimum and maximum number indexes also show a similar phenomenon. The reason is that with the increase of Δ*M* (*i.e.*, the increase of measurement required by each round of reconstruction (Δ*M*) also grows), according to the conclusion of Proposition 1, it is more difficult to meet the measurement termination condition under that situation. Another typical characteristic is that at the beginning of increasing Δ*M*, the SNR of reconstructed signal increases rapidly; but when the SNR reaches a certain level, further increasing Δ*M* has little effect. For example, for dataset OTD-A, when Δ*M* = 1, Min SNR = 22.9, and when Δ*M* = 4, SNR is up to 40.49; if continuing to increase Δ*M* to 8, the corresponding SNR becomes 43.5. This feature is caused by the shortcoming of CS reconstruction algorithm itself in processing non-strictly sparse signals, which is beyond the scope of this article. Note that, although we can acquire a higher recovery quality and a lower error rate (1 − 3^−Δ*M*^ according to Proposition 1) with a higher Δ*M* value, the measurement cost (constructed in the network) would also have a significant increase (because of the extra number of required measurements). So, on the whole, there is a tradeoff between the performance and the cost, and the specific network implementation target also should be considered to choose a proper value of Δ*M*.

### 6.3. Experiment of Data Gathered by Practical Network

**Figure 14:**
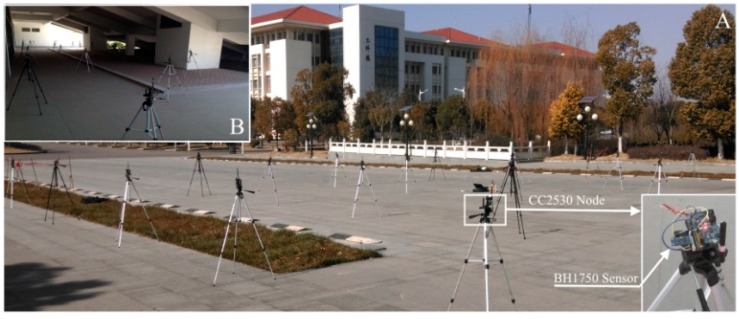
Network deployment of data gathering WSN. (**A**) Outdoor deployment; (**B**) Semi-outdoor deployment.

In order to evaluate the performance of the proposed algorithm in dealing with the data acquired from an actual environment, we implemented a WSN consisting of 30 nodes to monitor the luminosity of the environment. The microprocessor of the sensor nodes is CC2530 [23], and the luminosity-observation sensor is BH1750 [24]. The measurement resolution of this sensor is 0.1 (*lx*), and the measurement error is ±5 (*lx*) in the measurement range of 0 to 65,535 (*lx*). To test the impact of the collected signals on the proposed method under different experiment environments and topology conditions, we deployed the network into both outdoor and semi-outdoor scenes. The so-called semi-outdoor is as shown in Figure 14B, *i.e.*, a part of nodes are placed outdoors, and the rest are placed in the building. In order to obtain the entire environmental luminosity data, the sink node will enquire the sensor readings of all nodes every 5 min.

**Figure 15:**
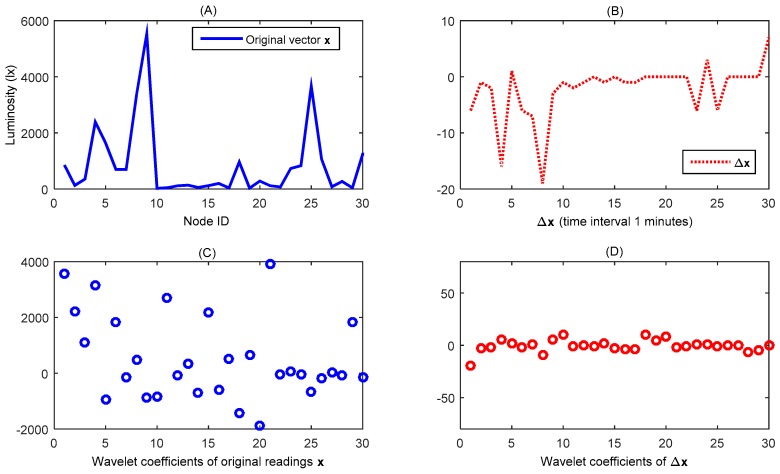
Wavelet representation of an original illumination data vector *x* and the difference vector Δ*x*. (**A**) Original data vector *x*; (**B**) Difference between two consecutive data vectors Δ*x*; (**C**) Wavelet representation of *x*; (**D**) Wavelet representation of Δ*x*.

According to our experimental results, our proposed schemes can be directly applied to both uniform and random outdoor deployment experiments; but for the semi-outdoor experimental data, the algorithm is not able to meet the stopping condition within the effective measurement steps. Analysis indicates that, in the semi-outdoor experiment, nodes placed outdoors are exposed to strong light, while their neighbor nodes may be placed in a relatively dimmed building, which will destroy the spatial sparse characteristics of the luminosity data observed by the whole network (for one epoch of the network sensed data (denoted as vector *x*) which is shown in Figure 15A, we can see that it is not sparse under wavelet transformation, as shown in Figure 15C).

After examining the statistics of the observation data, we find that, although the reading values of the adjacent nodes are very different, for the two consecutive rounds of network sensed data, there are a few differences between the readings of the adjacent nodes. We let $x_{i}^{t}$ denote the sensor reading of node *i* during data collecting epoch *t*; $\Delta x_{i}^{t}=x_{i}^{t+1}-x_{i}^{t}$ denotes the difference of node *i* between two consecutive periods. Then $\Delta x_{i}^{t}$, $\Delta x_{j}^{t}$ of the adjacent nodes should be related (the experimental results are shown in Figure 15B,D; the wavelet coefficient of *x* is emanative, but the wavelet transformation coefficient of Δ*x* obviously tends to be 0). If we denote $x_{t+1}$ and $x_{t}$ as the (*t* + 1)*th* and the (*t*)*th* network sensed data, we can acquire the following two equations:

(7) $$\Delta x^{t}=x^{t+1}-x^{t}$$

(8) $$\Delta x^{t}=\Psi U$$

We assume that the previous rounds of data collection have been completed, and the network sensed data $x^{t}$ has been worked out at the sink node. Thus, if the sink node has received the measurement results $y^{t+1}$ at *t* + 1 epoch, where $y^{t+1}=\Phi^{t+1}x^{t+1}$, from Equation (7), by solving out $\Delta x^{t}$, we can get $x^{t+1}$. Actually, $\Phi^{t+1}$ can be treated as the measurement matrix of $\Delta x^{t}$, because:

(9) $$y^{t+1}-y^{\prime t}=\Phi^{t+1}x^{t+1}-\Phi^{t+1}x^{t}=\Phi^{t+1}\Delta x^{t}$$

Therefore, by Equations (8) and (9), $\Delta x^{t}$ can be calculated through a typical CS reconstruction method. It should be pointed out that ${y^{\prime }}^{t}=\Phi^{t+1}x^{t}\neq y^{t}$ in Equation (9). This is because measurement matrices are randomly generated in different data gathering epochs.

With this method, we experiment on two consecutive rounds of data collected under each deployment condition. The experimental results are shown in Table 5. We can see that, with an increasing Δ*M*, both the recovery quality of signal and the required number of measurements increase. But for outdoor experimental data, SNR of reconstructed signal between Δ*M* = 5 and Δ*M* = 6 changes slightly; thus, considering the factor of the average number of measurements, for this application and the reconstruction algorithm, Δ*M* = 5 will be a good choice.

## 7. Conclusions

In this paper we have proposed a feedback-control-based compressed data gathering scheme. Different from existing studies, in this scheme, the sink node can adaptively adjust the measurement formation according to the reconstruction of received measurements in each data gathering epoch. To save the energy cost of the measurement-formation process in the network, a sparse measurement method and optimized measurement-formation construction strategy are proposed, and the control traffic on the network for requesting additional measurements is designed to combine with the generation of measurement coefficients. Simulation experiments verify that our measurement formation method outperforms existing methods in both energy consumption and controlling message quantity. Moreover, datasets from both OTD and practical network deployment also show the effectiveness of our proposed feed-back data recovery method.

## Figures and Tables

**Table 1:** Data reconstruction experiment on sparse Bernoulli measurement by using Proposition 1.

Δ*M*	Errors	Upper Bound	Average Measurement	Standard Deviation
1	128	200	31.0733	6.9430
2	2	200/3	34.8133	4.8461
3	0	200/9	36.7350	4.8882
4	0	200/27	37.9867	4.8428

**Table 2:** Parameters of the simulation experiments.

Parameter	Value
size of network	800 × 800
number of nodes	100~1200
max transmission radius	800$\sqrt{5}$/(number of nodes)
sink node position	(400,800)
packet length	120 Byte
PHY layer	Free space propagation model
MAC layer	CSMA/CA
E_elec	50 nJ/bit
E_start	250 nJ/packet
channel type	erasure channel

**Table 3:** Parameters of the OTD simulation experiments.

Parameter	Value
data file of OTD A	1403291948.cor
data file of OTD B	1404020436.cor
transformation base	6-level Daubechies2
reconstruction method	BP
experiment rounds	500
relative error	$\left\|\hat{x}-x\|/\|x\right\|$
SNR	$20\;\text{lg}\left(\left\|x\|/\|\hat{x}-x\right\|\right)$
range of Δ*M*	1~8

**Table 4:** Experiment result of our proposed method performed on OTD datasets.

Δ*M*	DataSet	Number of Measurements			Relative Error of Results			MIN SNR
		AVG	MIN	MAX	AVG	MIN	MAX	
1	A	143.43	103	198	0.1042	0.0139	0.4568	22.9357
	B	141.60	103	180	0.1202	0.0110	0.4504	22.2775
2	A	189.26	118	274	0.0225	0.0068	0.1527	34.1279
	B	181.07	156	210	0.0152	0.0056	0.0517	37.0925
3	A	222.04	160	283	0.0131	0.0069	0.0445	38.2235
	B	196.70	169	226	0.0102	0.0045	0.0197	40.3772
4	A	246	180	316	0.0098	0.0057	0.0175	40.4925
	B	211.73	176	244	0.0066	2.1694 × 10^−8^	0.0115	47.3546
5	A	256.85	175	330	0.0090	0.0051	0.0212	41.2457
	B	213.33	165	265	0.0065	4.6999 × 10^−10^	0.0144	48.6981
6	A	276.22	202	334	0.0078	0.0043	0.0135	42.3482
	B	225.20	196	262	0.0047	4.5934 × 10^−11^	0.0078	62.4401
7	A	286.69	219	338	0.0070	0.0043	0.0127	43.3284
	B	220.87	184	254	0.0052	6.7932 × 10^−11^	0.0118	64.2592
8	A	296.24	212	356	0.0069	0.0040	0.0132	43.4967
	B	225.07	188	268	0.0049	1.3999 × 10^−10^	0.0105	69.4010

**Table 5:** Experiment of reconstruction the data collected by the network.

Location	Topology	Δ*M* = 4		Δ*M* = 5		Δ*M* = 6	
		Measurement	SNR	Measurement	SNR	Measurement	SNR
outdoor	mesh	9.3	32.5	14.4	62.68	20.6	67.83
	random	12.4	28.6	15.0	42.35	22.5	42.72
semi-outdoor	mesh	13.3	32.1	17.9	31.3	24.4	46.06
	random	14.9	29.7	19.9	45.55	26.6	44.08
